# Electrochemical Performance and Time Stability of the Solid Oxide Cells with a (La,Sr)(Ga,Fe,Mg)O_3−δ_ Electrolyte and (La,Sr)(Fe,Ga,Mg)O_3−δ_ Electrodes

**DOI:** 10.3390/nano15120935

**Published:** 2025-06-16

**Authors:** Egor Gordeev, Ekaterina Antonova, Denis Osinkin

**Affiliations:** 1 Kinetics Laboratory, Institute of High-Temperature Electrochemistry, Ural Branch of the Russian Academy of Sciences, Yekaterinburg 620066, Russia; egorgordeev1998@mail.ru (E.G.); antonova_ek@list.ru (E.A.); 2 Scientific Laboratory of Electrochemical Devices and Materials, Institute of Hydrogen Energy, Ural Federal University, Yekaterinburg 620002, Russia; 3 Department of Life Safety, Institute of Fundamental Education, Ural Federal University, Yekaterinburg 620002, Russia; 4 Department of Environmental Economics, Graduate School of Economics and Management, Ural Federal University, Yekaterinburg 620002, Russia

**Keywords:** solid oxide fuel cell, symmetrical electrodes, chemical design, LSGM, LSF, DRT, long-term test

## Abstract

Electrochemical devices on solid electrolytes are closely considered from the point of view of efficient utilization of environmental resources in order to obtain a variety of products, including those with high added cost. This study provides insight into the functionality of electrochemical cells that have been designed with a specific configuration. These cells have the same ionic composition of the anode, cathode, and electrolyte. This was achieved by iron doping of highly conductive (La,Sr)(Ga,Mg)O_3−δ_ electrolyte, and gallium and magnesium doping of the electrode material based on (La,Sr)FeO_3−δ_. The main focus in this study is on the electrochemical behavior of such cells depending on the oxygen partial pressure in the gas phase, as well as the stability of the electrochemical performance over time for more than 950 h of testing. According to the obtained results, the electrochemical cell with a completely identical ionic composition of electrodes La_0.6_Sr_0.4_Fe_0.85_Ga_0.1_Mg_0.05_O_3−δ_ and electrolyte (La_0.8_Sr_0.2_)_0.98_Ga_0.7_Fe_0.1_Mg_0.2_O_3−δ_ demonstrated the best set of optimal performances. This consists of excellent chemical compatibility, high electrochemical activity (0.08 Ω cm^2^ in air at 800 °C), and a minor degradation rate.

## 1. Introduction

Electrochemical devices that utilize solid electrolytes possess a variety of distinctive characteristics. These devices facilitate direct conversion of chemical energy of the fuel into electricity in solid oxide fuel cells [1,2,3], conversion of gases under the electricity in solid oxide electrolyzers [4,5,6], production of high-purity gases [7,8,9], and gas detection using solid-state sensors [10,11,12], among numerous additional functions. The contemporary conventional technique for the design of such devices involves the implementation of a thick supporting electrode, predominantly composed of nickel-cermet, followed by the sequential deposition of the remaining layers (functional layer, electrolyte layer, protective layer, and cathode) on its surface [13,14]. This approach necessitates a substantial investment of time and energy, as numerous high-temperature firings are required to achieve the desired outcomes.

A novel approach to the design of electrochemical cells has recently emerged. This approach requires the implementation of a supporting electrolyte that exhibits high conductivity and electrodes that are structurally and chemically analogous, a concept called “symmetric cells” [15,16]. The fabrication of such cells is a relatively straightforward process. They do not require the prolonged reduction stage of the supporting fuel electrode during initial startup. Furthermore, these cells demonstrate a notable degree of tolerance to the redox cycling of both electrodes [17,18], a property that is frequently indispensable for processes such as carbon oxidation, which occurs during the combustion of hydrocarbon fuels.

In such cells, a highly conductive electrolyte based on lanthanum gallate [19,20] is used as an electrolyte and redox stable complex oxides, mainly based on strontium ferrite [21,22], are used as electrodes. At present, certain successes have been achieved in the development and research of such cells. For example, in [23], power densities of approximately 700 mW/cm^2^ at 800 °C were obtained using symmetric Sr_0.98_Fe_0.8_Ti_0.2_O_3−δ_-based electrodes on a LaGaO_3_-based electrolyte. As demonstrated in [24], a power density exceeding 800 mW/cm^2^ at 800 °C was achieved successfully through the utilization of symmetric Sr_2_Fe_1.5_Mo_0.5_O_6−δ_ electrodes and a symmetric Pr_2_NiO_4+δ_ catalyst. In [25], using an LSGM electrolyte with BaFe_0.9_Zr_0.1_O_3−δ_ electrodes, the authors obtained power densities of approximately 1100 mW/cm^2^ at 800 °C. In [26], the use of highly active Ba_0.5_Sr_0.5_Mo_0.1_Fe_0.9_O_3−δ_ electrodes in the cell with a supporting LSGM electrolyte resulted in the attainment of power densities of about 2280 mW/cm^2^ at 800 °C. The present values are of considerable power and are comparable to those of state-of-the-art conventional SOFCs based on a nickel–cermet anode [27].

The high redox stability of the electrodes is a key factor in the effective utilization of these cells in atmospheres with low oxygen partial pressure. Such atmospheres are formed at the cathode/electrolyte interface in fuel cells operating at high current densities, where oxygen uptake (electrochemical reduction) is elevated. Furthermore, a gradient of oxygen partial pressure may be formed in the electrode bulk, perpendicular to the electrolyte. This gradient may lead to the fact that the electrode surface in contact with the gas phase and the electrode layer in contact with the electrolyte may function at different oxygen partial pressures. In such a case, it is imperative to determine the kinetics of the oxygen reduction reaction at varying oxygen pressures with great precision.

The stability of the performance of high-temperature electrochemical devices is predominantly influenced by the stability of the electrodes over time. The underlying reason for this phenomenon is that the electrode is not merely a material in itself; rather, it is a thin, porous layer that is formed on a dense electrolyte. A number of processes that take place at elevated temperatures (segregation, sintering, vaporization, etc. [28,29,30,31,32,33]) make the electrode more susceptible to degradation over time compared to a dense electrolyte. Consequently, there is a necessity to develop not only active electrodes but also time-stable electrodes.

We have previously conducted research on the development of a highly conductive, triply modified LaGaO_3_-based electrolyte [34]. In this research, we proposed the concept of cation composition convergence between the electrolyte and electrodes. This convergence is intended to minimize chemical interaction, interdiffusion of cations, and convergence of thermo-mechanical compatibility. The objective of this study was to examine the electrochemical behavior of a series of (La,Sr)(Fe,Ga,Mg)O_3−δ_ electrode materials in contact with (La,Sr)(Ga,Fe,Mg)O_3−δ_ electrolytes as a function of temperature and oxygen partial pressure in a wide range of pO_2_ = 0.21–10^−21^ atm. Furthermore, this study involved the execution of long-term tests of electrochemical cells, with a duration of more than 500 h.

## 2. Materials and Methods

In this study, two types of electrolyte were investigated: La_0.8_Sr_0.2_Ga_0.8_Mg_0.2_O_3−δ_ (LSGM) and (La_0.8_Sr_0.2_)_0.98_Ga_0.7_Fe_0.1_Mg_0.2_O_3−δ_ + 0.5 wt.% Fe_2_O_3_ (LSGFM). The rationales underlying the selection of these electrolytes have been delineated in our previous research [34]. Electrolyte powders were obtained by the conventional solid state method utilizing La_2_O_3_ (99.9 wt.%, JSC “Vekton”, Russia, Saint Petersburg), SrCO_3_ (99.99 wt.%, JSC “Vekton”, Russia, Saint Petersburg), Ga_2_O_3_ (99.9 wt.%, JSC “Vekton”, Russia, Saint Petersburg), MgO (99.5 wt.%, JSC “Vekton”, Russia, Saint Petersburg), and Fe_2_O_3_ (99.9 wt.%, JSC “Vekton”, Russia, Saint Petersburg). The initial compounds were prepared according to the stoichiometric ratio and subsequently mixed in isopropanol. The mixture was then subjected to ball-milling in a planetary ball mill (Retsch^®^, Haan, Germany) at 250 rpm for a duration of 1 h. The obtained powder was subsequently subjected to a thermal treatment, namely annealing, at a temperature of 1000 °C for a duration of 6 h in an ambient atmosphere. This was followed by a grinding process in the planetary ball mill. In the case of the sintering aid (0.5 wt.% Fe_2_O_3_), it was incorporated into the powder and homogenized at 300 rpm for 1 h. To obtain dense ceramic samples, the powders were compressed into buttons by uniaxial cold pressing, followed by sintering at 1450 °C for 6 h in air.

The synthesis of electrode materials based on (La,Sr)FeO_3−δ_ was accomplished by using a self-propagating high-temperature synthesis technique. Citric acid was utilized as a complexing agent, with an amount that was 1.2 times the mass of the final compound. In the sequence of reactions, La_2_O_3_, SrCO_3_, Fe(NO_3_)_3_·9H_2_O (JSC “Vekton”, Russia, Saint Petersburg) (if necessary, MgO and Ga(NO_3_)_3_·8H_2_O (JSC “Vekton”, Russia, Saint Petersburg)), and C_6_H_8_O_7_·H_2_O (JSC “Vekton”, Russia, Saint Petersburg) were added to the glass beaker. The addition was made to an acidic nitric acid solution, which was stirred and maintained at a temperature of 80 °C. Note that each subsequent precursor was added to the reaction medium only after the previous one was completely dissolved. The solution was then evaporated on a hot plate at 340 °C until the combustion reaction was initiated. To complete the combustion reaction and remove solvent residues, the reactor was placed in a desiccator and maintained at 360 °C for 24 h. The resulting ash was ground in a mortar and subjected to a two-step high-temperature treatment at temperatures of 1000 and 1200 °C for 5 h in a muffle furnace (SNOL 6,7/1300, Tver, Russia). After each annealing, the powder was ground in a ball mill in isopropyl alcohol medium at 300 rpm for 1 h.

The symmetrical cells were fabricated by screen printing. The ink prepared from synthesized electrode materials with the addition of 3 wt.% Fe_2_O_3_ as a sintering additive was applied to the supporting electrolyte, followed by sintering at a temperature of 1050 °C for 2 h in a horizontal tube furnace (Yekaterinburg, Russia) with air purging. The compositions of the investigated electrochemical cells and their designation are shown in Table 1.

The X-ray powder diffraction analysis was performed using a D/MAX-2200 diffractometer (Rigaku Corporation, Takatsuki, Japan) with CuK_α_-radiation (λ(Kα) = 1.5406 Å, 40 kV, 30 mA) in ambient air at room temperature. The microstructure of the electrolyte/electrode boundary was analyzed by scanning electron microscopy (SEM) using a MIRA 3 (Tescan, Brno, Czech Republic) electron microscope.

The high-temperature studies were performed in a temperature range of 600–800 °C in horizontal tube furnaces in air and at various pO_2_ values from 0.21 to 10^−21^ atm. The extraction of oxygen from the ambient atmosphere was facilitated by electrochemical pumps based on stabilized zirconium oxide. The oxygen activity present in the gas phase was measured using a solid oxide oxygen sensor, which was placed near the samples that were the subject of study. The internal volume of the sensor was blown off with air. All measurements were carried out at atmospheric pressure. The electrochemical performance of the electrodes was investigated by using impedance spectroscopy, utilizing a Solartron FRA-1260 and EI-1287 (Ametek, Hampshire, UK). The distribution of relaxation time (DRT) analysis of the impedance spectra was performed using home-made original software based on Tikhonov’s regularization.

## 3. Results

### 3.1. XRD Certification

**Figure 1:**
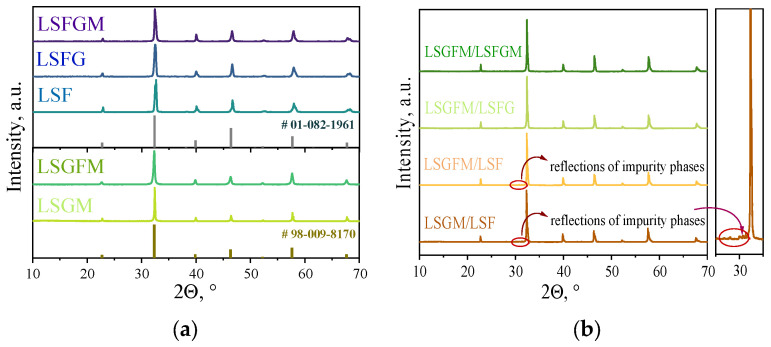
XRD patterns for investigated materials (**a**) and for composite powders after annealing at 1050 °C for 5 h in air (**b**).

In the initial step of the investigation, the phase compositions and chemical compatibility of the electrode and electrolyte materials were investigated. According to the results of the X-ray phase analysis, all the materials investigated are single phase (Figure 1a). Reflexes from impurity phases are absent from the X-ray patterns. Also, a slight shift of peaks can be seen in the X-ray diffraction patterns upon doping. This is due to changes in the crystal lattice parameters (due to the different radii of iron Fe^3+^ 0.55 Å and gallium Ga^3+^ 0.62 Å), which were previously observed in the literature in [35] for the LSGM electrolyte, and in [36], the authors noted an increase in the lattice parameter in LSF upon substitution of iron by gallium cations. The chemical compatibility tests of some electrolyte/electrode couples were carried out on composite powders consisting of a mixture of electrode and electrolyte powders in a mass ratio of 1:1. The composite powder was mixed in the planetary ball mill and calcined at 1050 °C for 5 h in air. According to the X-ray phase analysis data for LSGM/LSF and LSGFM/LSF couples, the appearance of low-intensity reflections is observed in the range of angles 30–32° (Figure 1b). These reflections have been assigned to the LaSrGaO_4_ and LaSrGa_3_O_7_ phases. Despite the fact that these phases have low conductivity [37], the intensity of the peaks indicates that they are formed in small amounts, about 2 wt.%. For this reason, these electrode/electrolyte pairs were also considered in further electrochemical studies.

### 3.2. Electrochemical Measurements

**Figure 2:**
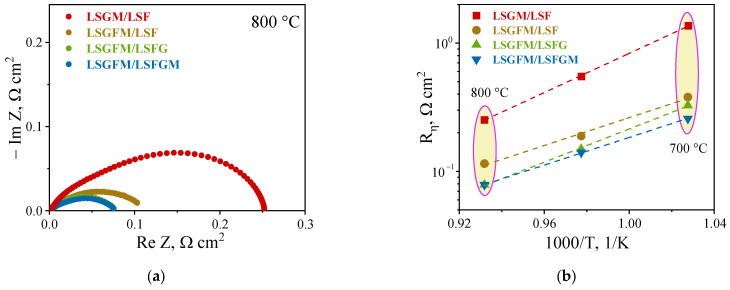
Impedance spectra (**a**) and temperature dependence of polarization resistances (**b**) of investigated electrochemical cell in air.

In the inception of high-temperature electrochemical studies, impedance spectra were measured in an air atmosphere. For illustrative purposes, Figure 2a presents the spectra at 800 °C. As is evident, the spectra manifest as a compressed half-circle with indistinct delineation into half-circles. Depending on the nature of the electrode and electrolyte, the spectra undergo negligible changes. However, for LSGM/LSF, a slightly pronounced separation of half circles is observed. The calculation of polarization resistance values was derived from the impedance spectra. The temperature dependences of these values are illustrated in Figure 2b. As demonstrated in Figure 2b, the dependences exhibit a linear form within the investigated temperature range of 700 to 800 °C. The activation energy values of the polarization resistance, calculated from the slope of the dependence, are in the range of 101–140 kJ/mol. Polarization resistance values at 800 °C were approximately 0.25, 0.11, 0.08, and 0.08 Ω cm^2^ for LSGM/LSF, LSGFM/LSF, LSGFM/LSFG, and LSGFM/LSFGM, respectively. The values obtained are satisfactory and consistent with the polarization resistance values of the doped ferrite-based electrodes (Table 2).

A close examination of the values of polarization resistance obtained for LSGM/LSF and LSGFM/LSF pairs is needed. Despite the identical electrode composition, the polarization resistance of the second cell was more than twice as low. This phenomenon can be attributed to the inherent properties of charge carriers within the supporting electrolyte. The introduction of iron into the gallium sublattice in LSGM results in the formation of a negligible number of electronic charge carriers. This, in turn, leads to the expansion of the electrochemical reaction area of oxygen reduction to the electrolyte surface. Consequently, there is a decrease in polarization resistance. This phenomenon is well documented and has been previously demonstrated by the present author in [44].

**Figure 3:**
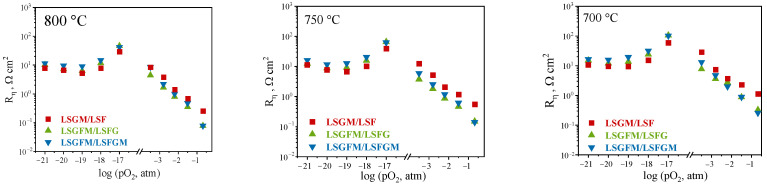
Concentration dependencies of polarization resistances at different temperatures.

Furthermore, the polarization resistance of the electrodes was investigated as a function of the partial pressure of oxygen in the gas phase at varying temperatures (Figure 3). Within the scope of this research, we have studied the behavior of three electrochemical cells LSGM/LSF, LSGFM/LSFG, and LSGFM/LSFGM. As can be seen, at all investigated temperatures, a close pattern of behavior of concentration dependences is observed for all samples. In the range of high oxygen partial pressures (pO_2_ = 0.21–10^−3.5^ atm), an increase in polarization resistance with decreasing pO_2_ is observed. This behavior is typical for complex oxides exhibiting mixed ion–electron conductivity and is associated with a decrease in the concentration of the potential-determining species in the gas phase, which in this case is oxygen. In the range of low oxygen partial pressures (pO_2_ = 10^−17^–10^−22^ atm), significant variations in the trends of change in polarization resistance are observed. The dependences exhibit extreme behavior, with a pronounced minimum at pO_2_ = 10^−19^ atm. This phenomenon is likely to be due to a modification in the conductivity characteristics of the electrodes, which is, in turn, probably precipitated by the partial reduction of iron within the regions of low oxygen partial pressures. This, in turn, results in a shift in the ratio of charge carrier concentrations. Furthermore, within this range of oxygen partial pressures, the mechanism of the electrochemical reaction varies significantly from changes (compared to an oxidizing atmosphere) in the nature of the electroactive components of the gas phase. In such cases, the potential determining components are hydrogen or CO, which are formed by the electrochemical decomposition of water and carbon dioxide, which are always present in ambient air. This alteration in the gas environment exerts an influence on the polarization resistance. Thus, at the transfer from air atmosphere to pO_2_ = 10^−3.5^ atm, the polarization resistance increases by approximately two orders of magnitude for all samples and is approximately 10 Ω cm^2^. The highest polarization resistances are obtained at pO_2_ = 10^−17^ atm and are approximately 100 Ω cm^2^. At a further decrease in pO_2_, the polarization resistance decreases, and at pO_2_ = 10^−19^ atm, it is in the region of 10 Ω cm^2^; at a further decrease in pO_2_, the polarization resistance increases again.

**Figure 4:**
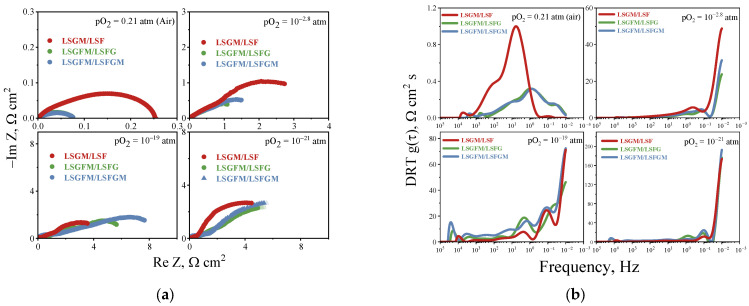
Impedance spectra (**a**) and DRT functions (**b**) for the investigated electrodes at 800 °C.

To achieve a more profound understanding of the electrode reaction mechanism and its sensitivity to oxygen partial pressure, it is imperative to examine the impedance spectra and DRT functions derived from these spectra. As illustrated in Figure 4a, the spectra of all investigated electrochemical cells are shown at varying pO_2_. Figure 4a demonstrates that the appearance of impedance spectra does not depend significantly on the chemical composition of the electrode, but it depends significantly on the partial pressure of oxygen in the gas phase. Thus, with decreasing oxygen content in the gas phase, the appearance of clearly distinguishable half-circles is observed. It can be seen that in an atmosphere with high oxygen partial pressure, the highest polarization resistance is characteristic of the LSGM/LSF cell, and with decreasing pO_2_, the polarization resistance of the LSGM/LSF cell becomes lower than that of the cells on the supporting LSGFM electrolyte.

Figure 4b illustrates the DRT functions calculated from the impedance spectra. The first thing to notice is the significantly different DRT functions for the LSGM/LSF cell and the other electrodes in air atmosphere. For the LSGM/LSF system, two distinct peaks are observed in the frequency range of 10 and 100 Hz, which are associated with the reactions of oxygen interphase exchange between the electrode and the gas phase (stages of dissociative adsorption and incorporation of oxygen into the crystal lattice of the electrode) [24]. For the other samples, a significant shift of the peak position to the low-frequency region is observed. This phenomenon is associated with the presence of electronic charge carriers in the electrolyte when iron cations are introduced into it and was considered by us earlier in [44]. It is worth noting that for electrodes in contact with LSGFM electrolyte, the type of DRT function does not change in an air atmosphere depending on the electrode composition, which indicates the same mechanism of the oxygen reduction reaction on LSFG and LSFGM electrodes. With decreasing oxygen partial pressure, the appearance of the DRT function changes significantly for all samples. The maximum peak begins to register in the region of the lowest frequencies. This behavior is associated with a significant contribution of gas diffusion resistance, which is due to the low content of electroactive components in the gas phase and a decrease in the total pressure in the measuring setup due to pumping out of oxygen. It is also worth noting that, with decreasing oxygen partial pressure there is a shift of peaks to the low-frequency region, especially in atmospheres with low oxygen partial pressure. This is due to the change of the electrode reaction mechanism from the oxygen reduction reaction to the oxidation reaction of hydrogen and CO, which remain in the gas medium after pumping oxygen out [24].

### 3.3. Long-Term Testing

It is imperative to consider the long-term stability of the electrochemical activity (polarization resistance) when assessing the properties of electrodes. To determine the stability of electrode performance, a series of long-term cell tests were conducted at a temperature of 800 °C for a duration exceeding 500 h. It should be noted that the long-term tests were performed on the same samples as those on which the oxygen partial pressure dependences were investigated. This approach is due to the fact that it is possible to observe not only the effect of time on electrode characteristics but also changes in the gas environment (including an atmosphere with extremely low oxygen content, pO_2_ = 10^−21^ atm).

**Figure 5:**
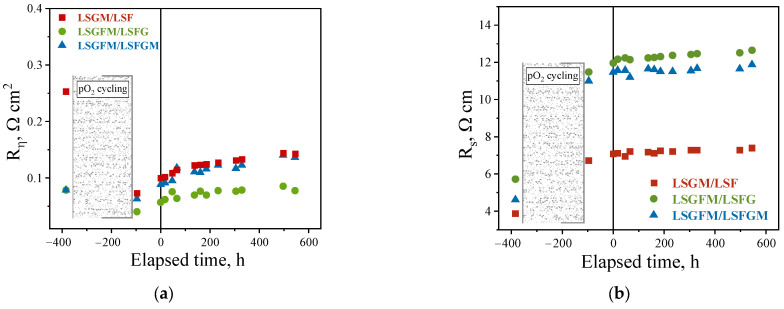
Time dependences of polarization resistance (**a**) and series resistance of cells (**b**) in air atmosphere at 800 °C.

In Figure 5, time dependences of the electrode polarization resistance and the ohmic resistance of the supporting electrolyte can be observed. The graphs are plotted in such a way that the OX scale starts from the negative region. The initial point pertaining to the dependence is derived from the temperature dependence depicted in Figure 2. Electrochemical measurements were conducted at varying oxygen partial pressures for a duration of approximately 300 h. Subsequently, the samples were transferred to an air atmosphere, and after a day of exposure, measurements were performed (corresponding to the point on the OX axis at −100 h). And then, after about 100 h, long-term tests were started (the first point corresponds to 0 h) for 500 h. Thus, the whole cycle of the experiment took more than 950 h.

As demonstrated in Figure 5a, the polarization resistance values after the measurement cycle at different oxygen partial pressures showed a decrease compared to the initial values. This phenomenon is attributed to the partial reduction of iron cations, a process that exerts a substantial influence on the electrochemical characteristics of the electrodes (as previously mentioned). This effect was most pronounced for LSGM/LSF cells. Consequently, for this particular cell, the electrode activity exhibited an approximate threefold increase prior to and after measurements from pO_2_. In contrast, for the other samples, this change was considerably less pronounced. It was found that, in general, all electrodes exhibited satisfactory stability of the polarization resistance during long-term testing. After a series of experiments totaling more than 950 h, including 300 h of experiments at different pO_2_ and temperatures, the resulting polarization resistance was found to be approximately 0.08, 0.14, and 0.15 Ω cm^2^ for LSGFM/LSFG, LSGFM/LSFGM, and LSGM/LSF cells, respectively (Figure 5a). Furthermore, the polarization resistance of these cells remained constant over the course of approximately 400 h, which is a highly promising indication.

Furthermore, the behavior of the series resistance of the investigated cells during long-term tests was examined. The series resistance is principally determined by the resistance of the supporting electrolyte and the resistance of the electrode/electrolyte interface. As demonstrated in Figure 5b, the series resistance of the cells exhibited a substantial increase after measurements of the oxygen partial pressure dependences. This increase persisted until the end of the long-term tests, at which point it did not revert to its initial value. This effect was most evident in samples with LSGFM electrolyte. This phenomenon appears to be attributable to the degradation of the electrolyte itself under conditions of very low oxygen partial pressures. Earlier observations of such rapid degradation in the presence of a humid hydrogen atmosphere have been documented in [34]. This behavior is characteristic of the LSGM electrolyte to a lesser extent. It is noteworthy that from the start of the long-term test report (0 h), the ohmic resistance of the cells remained constant, indicating the stability of the electrode/electrolyte interface.

**Figure 6:**
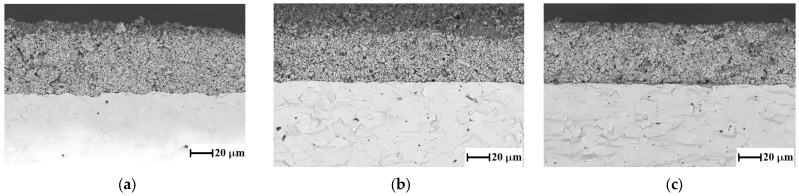
SEM images of investigated cells after 950 h of testing. LSGM/LSF (**a**), LSGFM/LSFG (**b**), and LSGFM/LSFGM (**c**).

Cross-sectional electrode/electrolyte SEM images obtained on the samples after 950 h of testing are shown in Figure 6. It can be seen that, in all cases, no defects are observed at the electrode/electrolyte interface, and all electrodes retain good porosity throughout the electrode thickness.

**Figure 7:**
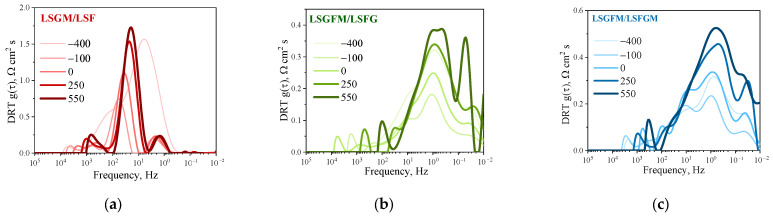
DRT functions during long-term test at 800 °C for LSGM/LSF (**a**), LSGFM/LSFG (**b**), and LSGFM/LSFGM (**c**) cells.

DRT functions were examined over a range of times during extended testing (Figure 7). It is evident that in the case of LSGM/LSF (Figure 7a), the DRT function before and after measurements as a function of pO_2_ (times −400 and −100 h) underwent significant changes in terms of peak intensities, although the positions and relaxation frequencies of the peaks remained constant. Subsequent tests revealed that the manifestation of the DRT function remained almost unchanged (times 0, 250, and 550 h). It has been demonstrated that when the oxygen partial pressure is modified, the LSF electrode undergoes irreversible alterations in relation to the oxygen reduction reaction mechanism. It is conceivable that the reduction atmosphere initiates the formation of active centers on the electrode surface (which may be additional oxygen vacancies), through which interfacial exchange with the gas phase is initiated, leading to an increase in the activity of the electrode. A similar pattern was observed for the other electrodes; however, the change in the intensity of the peaks before and after pO_2_-dependency studies was less pronounced. For LSGFM/LSFG and LSGFM/LSFGM cells, the appearance of the DRT function is much more complicated, which is due to the extension of the electrochemical reaction area to the electrolyte surface (as mentioned above). The abrupt change in the DRT function for the LSGFM/LSFG cell (Figure 7b) around 550 h of testing, namely, the split of the main peak and the appearance of a high-intensity peak in the low-frequency region, draws attention. Such behavior was not observed for other cells. For the LSGFM/LSFGM cell, a uniform insignificant increase in the intensity of all peaks is observed (Figure 7c), apparently due to the slow degradation processes that are always present in porous electrodes at high temperatures. The primary cause of this phenomenon is likely to be attributed to the delayed sintering of the electrode, which consequently results in a decrease in the number of active centers responsible for oxygen adsorption from the gas phase. This phenomenon can be substantiated by the increase in the intensity of the main peak over time, which is directly associated with the interphase exchange process between the electrode and the gas phase.

## 4. Conclusions

In this study, electrochemical cells on solid electrolytes with close and even identical ionic composition of the anode, cathode, and electrolyte were investigated. This was made possible by iron doping of highly conductive (La,Sr)(Ga,Mg)O_3−δ_ electrolyte and gallium and magnesium doping of the electrode material, based on (La,Sr)FeO_3−δ_. The results of the chemical compatibility study showed that the electrode/electrolyte material couples LSF/LSGM and LSF/LSGFM form impurity phases LaSrGaO_4_ and LaSrGa_3_O_7_ at co-sintering, while LSFG/LSGFM and LSFGM/LSGFM do not form impurity phases. It was found that, of all electrochemical cells studied, the LSFGM electrode in contact with the LSGFM electrolyte had the lowest polarization resistance of about 0.08 Ω cm^2^ at 800 °C in air. In the studies of electrochemical activity of electrodes depending on the oxygen partial pressure in the gas phase, it was found that the activity of all electrodes significantly decreased, even at a slight decrease in the oxygen content in the gas phase. In the range of very low oxygen partial pressures (pO_2_ = 10^−17^–10^−21^ atm), an extreme behavior of polarization resistance was observed, with a polarization resistance minimum of approximately 10^−19^ atm. Analysis of the impedance spectra by the distribution of relaxation times method showed different behavior of the electrode reaction in air atmospheres and in atmospheres with low oxygen partial pressure. This behavior was explained by the change in the electrode reaction mechanism and the nature of the potential-determining component in the gas phase, from oxygen in an air atmosphere to hydrogen and CO, in the case of gas atmospheres from which oxygen was pumped out to the residual pressure pO_2_ = 10^−21^ atm. It is shown that in an oxidizing atmosphere, the rate of the electrode reaction is determined by the stage of oxygen interphase exchange with the gas phase, whereas, at extremely low oxygen partial pressure, the gas diffusion stage begins to make a significant contribution to the polarization resistance of the electrode. Long-term tests were performed in an air atmosphere at 800 °C for more than 500 h (the whole cycle of measurements was more than 950 h) and showed good stability of the electrochemical activity of the electrodes over time. Scanning electron microscopy data showed that after a long cycle of all measurements, the microstructure of the investigated samples remained satisfactory, both in terms of electrode/electrolyte boundary quality and electrode porosity. According to the obtained results, the first produced and tested electrochemical cell with completely identical ionic composition of electrodes La_0.6_Sr_0.4_Fe_0.85_Ga_0.1_Mg_0.05_O_3−δ_ and electrolyte (La_0.8_Sr_0.2_)_0.98_Ga_0.7_Fe_0.1_Mg_0.2_O_3−δ_ demonstrated the best set of optimal performances. This consists of both excellent chemical compatibility, high electrochemical activity (0.08 Ω cm^2^ in air at 800 °C), and a minor degradation rate.

## Figures and Tables

**Table 1:** Investigated electrochemical cells and their designation.

Electrolyte Composition	Electrode Composition	Cell Designation
La_0.8_Sr_0.2_Ga_0.8_Mg_0.2_O_3−δ_	La_0.6_Sr_0.4_FeO_3−δ_	LSGM/LSF
(La_0.8_Sr_0.2_)_0.98_Ga_0.7_Fe_0.1_Mg_0.2_O_3−δ_ + 0.5 wt.% Fe_2_O_3_	La_0.6_Sr_0.4_FeO_3−δ_	LSGFM/LSF
	La_0.6_Sr_0.4_Fe_0.8_Ga_0.2_O_3−δ_	LSGFM/LSFG
	La_0.6_Sr_0.4_Fe_0.85_Ga_0.1_Mg_0.05_O_3−δ_	LSGFM/LSFGM

**Table 2:** Polarization resistances of ferrite-based electrodes at 800 °C in air.

Electrode	R_η_, Ω cm^2^	Ref.
Sr_2_Fe_1.5_Mo_0.5_O_6−δ_	0.24	[38]
LaSr_2_Fe_2_CrO_9−δ_	0.29	[39]
Sr_2_Fe_1.4_Nb_0.1_Mo_0.5_O_6−δ_	0.1	[40]
La_0.6_Sr_0.4_Co_0.2_Fe_0.6_Nb_0.2_O_3−δ_	0.36	[41]
La_0.9_Ca_0.1_Fe_0.9_Nb_0.1_O_3−δ_	0.24	[42]
La_1.4_Sr_0.6_FeO_4−δ_	5.82	[43]
La_0.6_Sr_0.4_FeO_3−δ_ (on LSGM)	0.25	This study
La_0.6_Sr_0.4_FeO_3−δ_ (on LSGFM *)	0.11	
La_0.6_Sr_0.4_Fe_0.8_Ga_0.2_O_3−δ_	0.08	
La_0.6_Sr_0.4_Fe_0.85_Ga_0.1_Mg_0.05_O_3−δ_	0.08	

*—discussion is provided below.

## Data Availability

The original contributions presented in the study are included in the article. Further inquiries can be directed to the corresponding author.
